# Cucurbitacin E and I target the JAK/STAT pathway and induce apoptosis in Sézary cells

**DOI:** 10.1016/j.bbrep.2020.100832

**Published:** 2020-10-15

**Authors:** Isabella J. Brouwer, Jacoba J. Out-Luiting, Maarten H. Vermeer, Cornelis P. Tensen

**Affiliations:** Department of Dermatology, Leiden University Medical Center, Leiden, the Netherlands

**Keywords:** Cucurbitacin, Cutaneous T-Cell lymphoma (CTCL), Sézary syndrome, JAK-STAT pathway, STAT3, Apoptosis

## Abstract

Cutaneous T-cell lymphomas and leukemias (CTCLs) are a heterogeneous group of extranodal non-Hodgkin's lymphomas. These are characterized by an accumulation of malignant CD4^+^ T-lymphocytes in the skin, lymph nodes, and peripheral blood. Novel treatment options are needed for patients who progress to advanced stage disease. Cucurbitacin I has previously shown promising results in Sézary syndrome (Sz). A plethora of cucurbitacins, however, have not yet been tested in CTCL. Herein, we investigated the effect of cucurbitacin E and I in two CTCL cell lines. We show that both cucurbitacins decrease viability and cause apoptosis in these cell lines, although HuT-78 was more affected than SeAx (IC_50_ of 17.38 versus 22.01 μM for cucurbitacin E and 13.36 versus 24.47 μM for cucurbitacin I). Moreover, both cucurbitacins decrease viability of primary cells of a Sz patient (56.46% for cucurbitacin E and 59.07% for cucurbitacin I). Furthermore, while JAK2 inhibition leads to decreased viability in SeAx cells (IC_50_ of 9.98 and 29.15 μM for AZD1480 and ruxolitinib respectively), both JAK1 and JAK3 do not. This suggests that JAK2 has a preferential role in promoting survival. Western blotting in SeAx cells revealed that both cucurbitacins inhibit STAT3 activation (P < 0.0001), while only cucurbitacin I inhibits STAT5 activation (P = 0.05). This suggests that STAT3 plays a preferential role in the mechanism of action of these cucurbitacins. Nevertheless, a role of STAT5 and JAK2 cannot be excluded and should be explored further. This knowledge could contribute to the development of effective therapies for CTCL and other malignancies involving dysfunction of the JAK/STAT pathway.

## Highlights

• Cucurbitacin E and I decrease viability and cause apoptosis in Sézary cell lines
• Both cucurbitacins decrease viability of primary cells of a Sézary patient
• STAT3 appears to play a role in the mechanism of action of cucurbitacin E and I

## Graphical abstract

**Figure:**
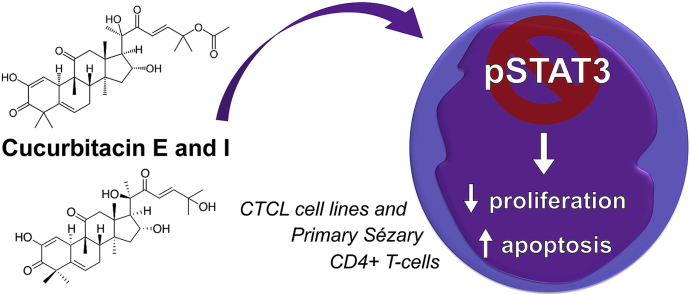

## 1 Introduction

Cutaneous T-cell lymphomas and leukemias (CTCLs) are a heterogeneous group of extranodal non-Hodgkin's lymphomas. These are characterized by an accumulation of malignant CD4^+^ T-lymphocytes in the skin, lymph nodes, and peripheral blood [[1], [2], [3], [4]]. The two major types of CTCL are mycosis fungoides (MF), which is restricted to the skin, and a more aggressive leukemic variant called Sézary syndrome (Sz) [5]. Despite an annual incidence of approximately 0.5 per 100,000, CTCL is a debilitating and incurable disease [[6], [7], [8], [9]].

Since CTCL is incurable, the focus in the treatment of CTCL is rather on preventing progression. Most patients suffering from early-stage disease have a favorable prognosis, however, 25% of these patients progress to advanced stages [10]. Advanced stage CTCL has a poor prognosis and survival of about 3 years [[10], [11], [12], [13], [14]]. Treatment options for advanced disease include systemic treatments, such as corticosteroids, extracorporeal photopheresis and immunotherapy [1,8,[15], [16], [17]]. Most of these systemic treatments only have partial response rates and do not provide a long-lasting therapeutic option [18]. Allogeneic bone marrow transplant could provide a long-lasting response in advanced patients, but has a high mortality and relapse rate [[18], [19], [20]]. Thus, regardless of the treatment options available, CTCL remains difficult to treat. One of the reasons for this difficulty is the lack of knowledge regarding its pathogenesis.

Abnormal janus kinase (JAK) and signal transducer and activator of transcription (STAT) protein signaling has been shown to be involved in the pathogenesis of CTCL. In Sz, mutations in JAK or STAT, amplification of DNA regions encoding STAT3/5, and epigenetic alterations in this signaling pathway have been found [16,[21], [22], [23], [24]]. Furthermore, in MF, deletions of genes encoding proteins which regulate negative feedback of the JAK/STAT pathway have been found [25,26]. These abnormalities all lead to constitutive STAT3/5 activation. Consequently, CTCL cells are very sensitive to treatment with STAT inhibitors, making this an interesting pathway to target [[27], [28], [29], [30], [31]]. Cucurbitacins could be promising compounds to target the JAK-STAT pathway.

Cucurbitacins are a family of plant-derived triterpenoids. Cucurbitacins have been reported to inhibit cancer cell proliferation through interference with the JAK-STAT pathway [[32], [33], [34]]. Based on their side-chain variations, cucurbitacins can be grouped in 12 main categories with different structures [35,36]. Interestingly, the inhibitory activity of cucurbitacins on the JAK/STAT pathway is dependent on the structure of the molecule. In structure-activity studies it was shown that cucurbitacin Q inhibits the activation of STAT3, cucurbitacin A inhibits JAK2, and cucurbitacins B, E, and I, inhibit the activation of both [33,37]. This suggests that different cucurbitacins could have different cytotoxic effects against CTCL.

Cucurbitacin I is the only cucurbitacin that has been tested in CTCL [32]. The compound caused a time- and concentration-dependent decrease of total and activated STAT3 in a Sz cell line and total STAT3 in freshly isolated Sz cells. Cucurbitacin I also caused 73–91% apoptosis in freshly isolated Sz cells [32]. Although these results are promising, the precise mechanism of the observed effects on CTCL is still unknown.

Elucidating the mechanism of action of cucurbitacins could lead to novel treatment options, with improved efficacy and limited side effects. In this study we investigated a selection of naturally occurring cucurbitacins to establish their effect on proliferation and apoptosis in two CTCL cell lines. Hereby, we aimed to further elucidate the effect of cucurbitacins on STAT3 and STAT5 signaling in CTCL and the role of JAKs. This could lead to the discovery of novel treatment options for CTCL, but also for other lymphomas and malignancies with JAK/STAT pathway activation.

## 2 Methods

### 2.1 Cell lines

The Hut-78 cell line (ATCC number TIB-161), derived from the peripheral blood of a patient with Sz [39] was cultured in RPMI-1640 GlutaMAX™ medium (Invitrogen, Breda, The Netherlands), supplemented with 10% fetal calf serum (HyClone/Greiner, Nürtingen, Germany), 100 IU ml^−1^ penicillin and 100 μg/ml streptomycin (Invitrogen). The SeAx line, also derived from the peripheral blood of a patient with Sz [38], was cultured in the same conditions, with the addition of 200 IU ml^−1^ IL-2 (PeproTech, Rocky Hill, NJ).

### 2.2 Study compounds

Cucurbitacin E (NSC 106399, Sigma-Aldrich, Saint Louis, MO), cucurbitacin I (NSC 521777, Indofine Chemical Company, Hillsborough, NJ) and Stattic (Calbiochem, San Diego, CA) were dissolved in DMSO to a concentration of 15 mM, 50 mM and 100 mM respectively. JAK inhibitors (JAKi) AZD1480 (Tocris, Bristol, UK), decernotinib (MedChem Express, Monmouth Junction, NJ), ruxolitinib (Selleck, Houston, TX) and tofacitinib (Pfizer, Capelle a/d Ijssel, The Netherlands) were dissolved in DMSO to a concentration of 10 mM. All compounds were stored at −20 °C. During the experiments, compounds were diluted in culture medium to the final concentrations.

### 2.3 WST-1 cell proliferation assay

Hut-78 and SeAx cells were plated in a flat-bottom 96-wells plate at a cell density of 50,000 cells per well (in 100 μL). Cucurbitacins and JAKi's were added to the wells for 48 h at various concentrations. Medium containing an equivalent volume of DMSO to the highest concentration of the compound was used as a control, this amounted to 0.5% DMSO. After 48 h, WST reagent (Roche, Woerden, The Netherlands) was added and quantitated with a scanning multiwell spectrophotometer by measuring absorbance at 450 nm and 650 nm after 0 and 6 h. The WST-1 assay is based on the cleavage of the tetrazolium salt to formazan by cellular mitochondrial dehydrogenase. The number of living cells is directly proportional to the amount of the dye generated by the activity of dehydrogenase. The percentage of viable cells in each treatment condition was determined by calculating the ratio of the absorbance in the treated wells to the non-treated wells. In the primary Sz cells assay both cucurbitacins were added at a concentration of 30 μM to primary cells from one Sz patient. Patient PBMCs were enriched for CD4^+^ T cells by negative selection using magnetic beads (CD4^+^ T-cell isolation kit, Miltenyi Biotec, Bergisch Gladbach, Germany) as described by van Kester et al. [32].

### 2.4 Cytotoxicity assay

SeAx cells were cultured in 96-well plates at a cell density of 50,000 cells per well for 24 h. Wells were coated in poly-l-ornithine (Sigma-Aldrich) according to the protocol provided by BioTek (Winooski, VT). Cucurbitacin E and I were added at a concentration of 20 μM. Medium containing an equivalent volume of DMSO was used as a control, this amounted to 0.3% DMSO. To visualize cell nuclei, a Hoechst staining (ThermoFisher, Waltham, MA) was performed. This staining was used to count the total number of cells at each time point. CellEvent™ Caspase-3/7 Green Detection Reagent (ThermoFisher) was added at a concentration of 5 μM to visualize the number of apoptotic cells at each time point. To calculate the percentage of apoptotic cells, the number of cells positive for the apoptotic staining was divided by the total number of cells positive for the Hoechst staining. Cells were visualized every 4 h by the Cytation™ 5 (BioTek), using phase contrast, DAPI and GFP standard filters. The Cytation™ 5 is an automated digital microscope and incubator, which allows for cells to be incubated while simultaneously visualizing processes such as apoptosis over time. Data were analyzed using the Gen5™ software (BioTek).

### 2.5 Western blotting

SeAx cells were plated in a flat-bottom 12-wells plate at a cell density of 1,000,000 cells per well. AZD1480, cucurbitacin E, cucurbitacin I and Stattic were added to the wells at a concentration of 10 μM, 20 μM, 20 μM and 4 μM respectively. Medium containing an equivalent volume of DMSO to the highest concentration was used as a control, this amounted to 0.3% DMSO. After 3 h, cells were lysed with lysis buffer containing RIPA buffer 10X (#9806), Phosphatase Inhibitor Cocktail 100X (#5870) and Protease Inhibitor Cocktail 100X (#5871), all purchased from Cell Signaling Technology (CST, Danvers, MA). Protein concentration was determined with a detergent compatible protein assay (Bio-Rad) and was used to load 15 μg of protein per sample. Electrophoresed protein was blotted to polyvinylidine difluoride membranes (Amersham Biosciences, Buckinghamshire, UK) overnight at 4 °C. After blotting, the membranes were blocked for 1 h at room temperature using a blocking buffer (LI-COR Biosciences, Lincoln, NE). Subsequently, the membranes were incubated overnight at 4 °C with STAT3 (#8768), *P*-STAT3^Tyr705^ (#9131), STAT5A/B (#94205) and *P*-STAT5^Tyr694^ (#9351) (all from CST). Rabbit anti-human glyceraldehyde 3-phosphate dehydrogenase (GAPDH, #5174, CST) was used as a loading control. Afterwards, the membranes were washed and incubated for 1 h with goat anti-rabbit IgG HRP (sc-2004, Santa Cruz Biotechnology, Dallas, TX). All antibodies were diluted in blocking buffer. Chemiluminescence (West Femto Maximum Sensitivity Substrate, Pierce, Rockford, IL) was used for visualization.

### 2.6 Statistical analysis

All experiments were performed in triplicate unless stated otherwise. Differences were considered significant at P < 0.05. Data are expressed as mean ± SD. Statistical analysis was performed using Graphpad Prism 5.0 (San Diego, CA). Nonlinear regression was used to determine IC_50_ values in the cell line WST-1 assays and one-way ANOVA was used to assess the significance of differences. WST-1 cell proliferation assay in primary Sz cells was performed once, with technical replicates of five wells per condition. Differences were assessed using one-way ANOVA. Linear regression was used to assess the significance of differences of the cytotoxicity assay. Protein levels in the Western blot experiments were quantified using ImageJ software (National Institutes of Health, Bethesda, MD), corrected for GAPDH loading control and normalized to their respective medium control. One-way ANOVA was used to assess the significance of differences.

## 3 Results

### 3.1 Cucurbitacin E and I inhibit proliferation in CTCL cell lines

**Fig. 1:**
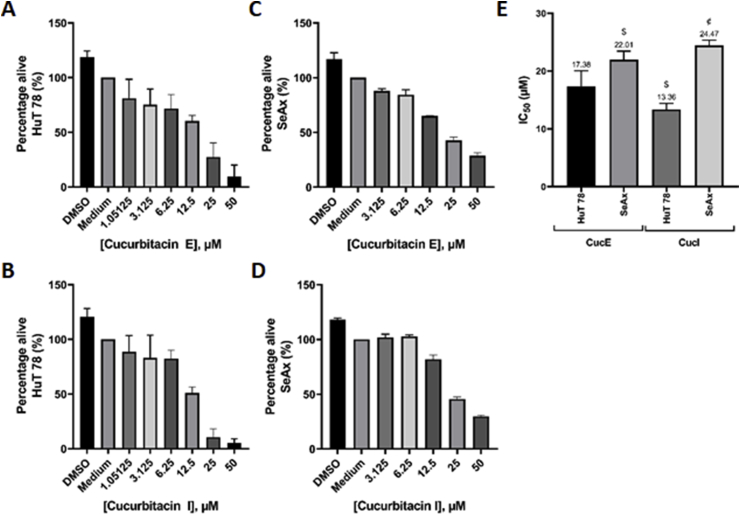
**Cucurbitacin E and I inhibit proliferation of HuT 78 and SeAx.A–D:** HuT 78 (A, B) and SeAx (C, D) cells were seeded at a cell density of 50,000 cells per well in a 96 wells plate. After incubation for 48 h with cucurbitacin E and I, viability was quantitated. **E:** The IC_50_ values for cucurbitacin E and I in both cell lines were determined. Error bars represent SD of three separate experiments. ^$^P < 0.05 vs cucurbitacin E HuT 78. ^¢^P < 0.0001 vs cucurbitacin I HuT 78.

After incubation for 48 h with cucurbitacin E and I, both cucurbitacins showed a dose-dependent anti-proliferative effect in HuT 78 and SeAx cell lines (Fig. 1). The IC_50_ values for cucurbitacin E were 17.38 and 22.01 μM and the IC_50_ values for cucurbitacin I were 13.36 and 24.47 μM for the HuT 78 and SeAx cell lines respectively. The IC_50_ values of cucurbitacin E and I in the HuT 78 cell line were significantly lower than the respective values in the SeAx cell line. Furthermore, within the HuT 78 cell line, cucurbitacin I had a significantly smaller IC_50_ value. In the SeAx cell line a trend could be seen towards cucurbitacin E having a higher cytotoxicity (P = 0.068).

### 3.2 Cucurbitacin E and I cause apoptosis in SeAx cells

**Fig. 2:**
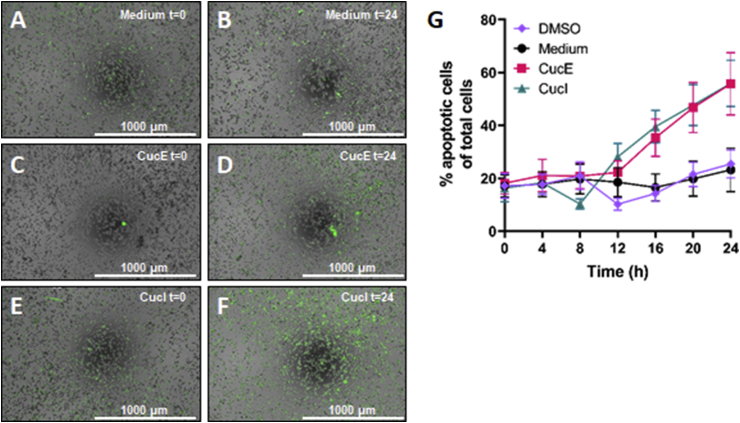
**Cucurbitacin E and I cause apoptosis of SeAx cells.** SeAx cells were cultured in the presence of cucurbitacin E and I at a cell density of 50,000 cells per well for 24 h. Every 4 h, the cells were visualized by the Cytation™ 5 (BioTek) using phase contrast, DAPI and GFP standard filters. **A, C, E:** Cells were visualized at t = 0. A, C and E represent the medium, cucurbitacin E and cucurbitacin I condition respectively. **B, D, F:** Cells were visualized at t = 24. A, C and E represent the medium, cucurbitacin E and cucurbitacin I condition respectively. **G:** The percentage of apoptotic SeAx cells was quantitated at each time point during 24 h. Error bars represent SD of two separate experiments.

To confirm whether cucurbitacin E and I induce apoptosis of SeAx cells, the Cytation™ 5 (BioTek) was used to visualize activation of caspase-3/7 in apoptotic cells. After 24 h, the images captured at the different time points were compared (Fig. 2A–F). While the medium and DMSO control showed almost no increase in the relative amount of apoptotic cells when gauged by eye, both cucurbitacin conditions showed an increase in apoptotic cells after 24 h as compared to baseline. Gen5™ software was used to quantitatively determine the amount of apoptotic cells at each time point (Fig. 2G). The level of apoptosis in the control conditions stayed relatively constant. Both cucurbitacins significantly increased the level of apoptosis (P < 0.001) to a similar degree as compared to the control conditions.

### 3.3 JAK1 and JAK3 inhibition do not induce an anti-proliferative effect in SeAx cells

**Fig. 3:**
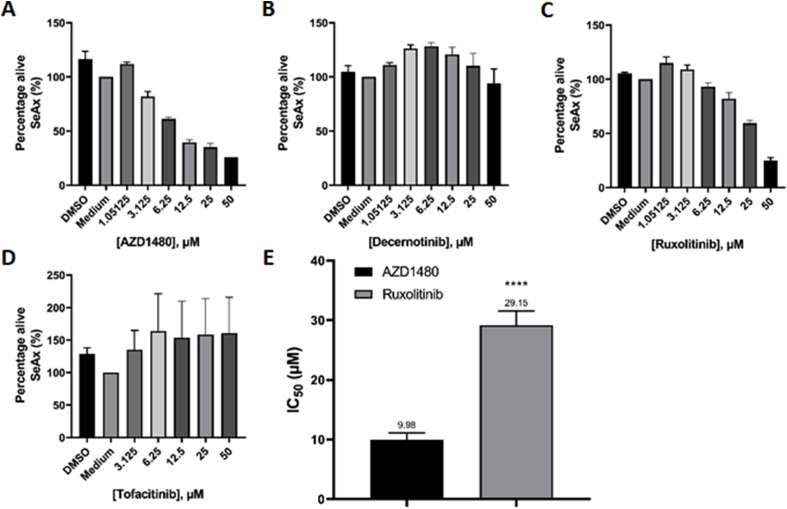
**JAK2 inhibitors inhibit proliferation, while JAK1/3 inhibitors do not. A-D:** SeAx cells were seeded at a cell density of 50,000 cells per well in a 96 wells plate. After incubation for 48 h with AZD1480, decernotinib, ruxolitinib and tofacitinib, viability was quantitated. **E:** IC_50_ values were determined for AZD1480 and ruxolitinib. Error bars represent SD of three separate experiments. ****P < 0.0001 versus AZD1480.

To investigate the effect of JAK inhibition on CTCL cells, SeAx cells were treated with a selection of JAKi's, including AZD1480, decernotinib, ruxolitinib and tofacitinib. AZD1480 has previously been described to inhibit JAK2, decernotinib is a specific JAK3 inhibitor, ruxolitinib inhibits JAK1 and JAK2 and tofacitinib inhibits JAK1 and JAK3 [[40], [41], [42], [43], [44]]. SeAx cells were first treated with these compounds at concentrations ranging from 5 nM to 1 μM, based on effective doses reported in the literature [44]. At these concentrations, WST-1 cell proliferation assay showed no effect (see Supplementary Fig. S1). To investigate whether these JAKi's truly have no effect on SeAx cells, a higher range of concentrations was chosen and WST-1 assays were performed (Fig. 3). At these higher concentrations, AZD1480 and ruxolitinib showed a dose-dependent anti-proliferative effect, with IC_50_ values of 9.98 and 29.15 μM respectively. In contrast, decernotinib and tofacitinib did not show any significant anti-proliferative effect.

### 3.4 Cucurbitacin E and I inhibit STAT3, but only cucurbitacin I inhibits STAT5

**Fig. 4:**
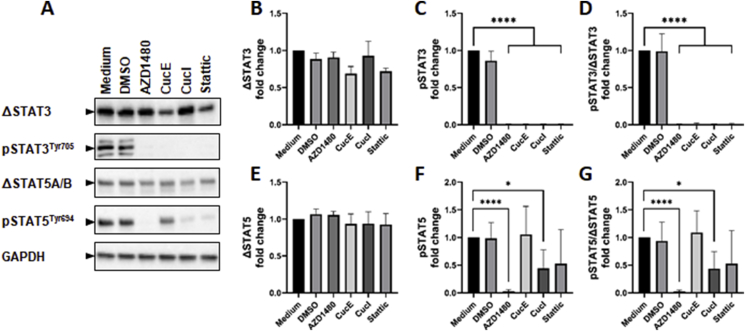
**Cucurbitacin I inhibits STAT3 and STAT5 activation, while cucurbitacin E only inhibits STAT3 activation. A:** Protein lysates of SeAx cells after treatment with AZD1480, cucurbitacin E, cucurbitacin I and Stattic were visualized to show the levels of STAT3, activated STAT3, STAT5A/B and activated STAT5. **B-G:** Protein levels were quantified, corrected for GAPDH loading control, and normalized to the respective medium control. Total STAT3 (B), pSTAT3 (C), pSTAT3/total STAT3 levels (D) and total STAT5 (E), pSTAT5 (F) and pSTAT5/total STAT5 levels (G) were determined. Error bars represent SD of either two (STAT3) or three (STAT5) separate experiments. *P = 0.05. ****P < 0.0001.

Previous studies have shown that curcurbitacin I can inhibit STAT3 in SeAx cells and primary Sz cells [32]. Moreover, cucurbitacin B and E have been shown to inhibit STAT5 activation in other cell types [45,46]. To investigate the role of STAT3 and STAT5 activation in SeAx cells treated with cucurbitacin E and I, levels of total and phosphorylated protein were measured (Fig. 4). Cells were also treated with the JAK2 inhibitor AZD1480 and Stattic, which selectively inhibits STAT3 dimerization relative to other STAT family members. Western blotting revealed that while AZD1480, cucurbitacin E, cucurbitacin I and Stattic all significantly downregulated the levels of activated STAT3, only AZD1480 and cucurbitacin I significantly downregulated the levels of activated STAT5. The effect on activated STAT5 caused by AZD1480 was to a similar extent to its effect on activated STAT3, however, the effect of cucurbitacin I on activated STAT5 was notably less pronounced. While none of the study compounds significantly affected total STAT3 levels, both cucurbitacin E and Stattic showed a trend towards a slight decrease of these levels.

### 3.5 Cucurbitacin E and I decrease viability of primary sz cells

**Fig. 5:**
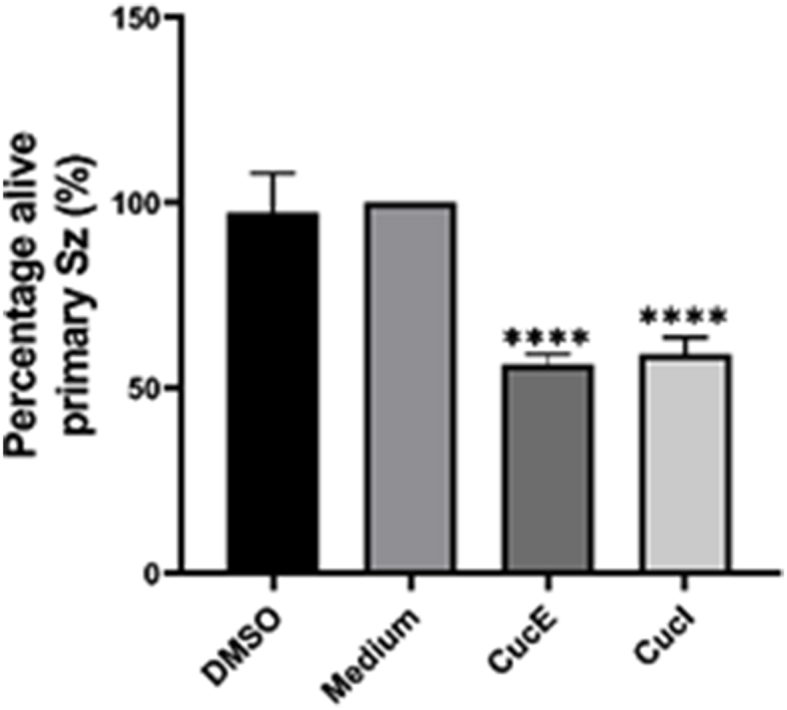
**Cucurbitacin E and I decrease viability of primary Sz cells.** Primary Sz cells of one patient were seeded at a cell density of 50,000 cells per well in a 96 wells plate. After incubation for 48 h with 30 μM cucurbitacin E and I, viability was quantitated. Error bars represent SD of technical replicates of five wells. ****P < 0.0001 versus medium.

To examine whether cucurbitacin E and I affect the viability of primary Sz cells, CD4^+^ T-cells isolated from a Sz patient were incubated with 30 μM cucurbitacin E and I (Fig. 5). After incubation, viability was 56.46% and 59.07% for cucurbitacin E and I respectively. Viability was not affected by medium containing volume equivalents of DMSO. Viability in both the cucurbitacin E and I condition was significantly different from the medium control (P < 0.0001).

## 4 Discussion

In this paper, we demonstrate that cucurbitacin E and I induce apoptosis in CTCL cell lines and decrease viability of primary Sz cells. We propose that this is achieved through inhibition of STAT3.

Previous research has found that cucurbitacins can inhibit cell proliferation in several cell lines [32,34,[47], [48], [49], [50], [51]]. We found that both cucurbitacin E and I inhibit proliferation of HuT 78 and SeAx cells in a dose-dependent manner. The IC_50_ values were significantly lower in HuT 78 than SeAx. Furthermore, cucurbitacin I affected HuT 78 cells more than cucurbitacin E, while cucurbitacin E showed a trend towards higher cytotoxicity in the SeAx cell line. These findings suggest that the change of the terminal OH group in cucurbitacin I to an alkoxy group in cucurbitacin E impacts its cytotoxicity.

The cytotoxicity of the cucurbitacins also appears to depend on the treated cell line. This variance has previously been observed in studies with non-CTCL cell lines, in which both the choice of cucurbitacin and of cell line were shown to affect cytotoxicity [50,52]. Interestingly, we found that both CTCL cell lines were affected differently by the cucurbitacins. Since HuT 78 and SeAx are derived from different patients and show distinct underlying mutations, this difference is credible [[53], [54], [55]]. Similarly to these cell lines, CTCL patients are not identical and neither are the mutations underlying their disease. In medical practice, this could mean that individual patients would benefit differently from the various cucurbitacins, suggesting a role for personalized medicine. This hypothesis is in line with previous research in which personalized medicine has been proposed to be required to properly treat individual patients, since CTCL displays heterogeneous phenotypes and differentiation profiles [[56], [57], [58]].

To further confirm these results, we investigated the ability of cucurbitacin E and I to induce apoptosis in SeAx cells. Cucurbitacin I has previously been shown to induce apoptosis in Sz patient cells [32]. We found that both cucurbitacins induce apoptosis to a similar extent. In the WST-1 assay, however, cucurbitacin E appeared to have a higher cytotoxicity. This discrepancy could be explained by the difference in incubation time of the two assays. Alternatively, the two cucurbitacins might affect proliferation and apoptosis differently, thereby explaining the difference between the two assays. Moreover, both cucurbitacins only showed a significant increase in the level of apoptosis after 16 h. This was expected, since we measured apoptosis by measuring activated caspase 3 and 7, which are activated in the mid-stage of apoptosis [59].

The cytotoxic effect of cucurbitacins in CTCL has previously been suggested to be mediated through the JAK-STAT pathway. Cucurbitacins have been proposed to affect mainly JAK2 and STAT3 [32,33]. In contrast to this, most mutations in CTCL found in the JAK family of proteins affect JAK1 or JAK3 [22,56]. Surprisingly, we found that only two of the four selected JAKi's in our study showed an anti-proliferative effect. These two compounds, AZD1480 and ruxolitinib, both inhibit JAK2 [[41], [42], [43]]. In contrast, the other two JAKi's, decernotinib and tofacitinib, do not inhibit JAK2; the former inhibiting JAK3 [40] and the latter JAK1 and JAK3 [44]. This finding suggests that JAK2 plays an important role in the effect of cucurbitacins in this CTCL cell line, while JAK1 and JAK3 do not.

The downstream targets of JAK2, the STAT proteins, have also been implied in both the pathogenesis of CTCL and the mechanism of action of cucurbitacins [27,29,30,[32], [33], [34]]. Van Kester et al. [32] previously showed that STAT3 activation and total STAT3 levels are affected by cucurbitacin I treatment in Sz. Furthermore, several cucurbitacins have been shown to inhibit STAT5 activation in other diseases [45,46]. This is interesting, since it has been suggested that STAT5, not STAT3, has an important role in promoting survival in lymphoid tumors [60]. To investigate the role of STAT3 and STAT5 in the mechanism of action of cucurbitacins in the SeAx cell line, we performed a Western blot experiment. We found that both cucurbitacin E and I inhibit the activation of STAT3, but we did not see a decrease of total STAT3 as a result of treatment with cucurbitacin E and I. This discrepancy with the study of Van Kester et al. [32] might be due to the difference in treatment time; 4 h might be an insufficient amount of time to observe an effect on total STAT3 levels. In contrast to activated STAT3, which was affected by both cucurbitacins, activated STAT5 levels were only significantly decreased by cucurbitacin I. This effect of cucurbitacin I on activated STAT5 was also notably less pronounced than on activated STAT3. This suggests a preferential role of STAT3 inhibition in the mechanism of action of cucurbitacins in the SeAx cell line, although a role for STAT5 inhibition cannot be excluded.

While STAT3 appears to play a preferential role to STAT5 in the mechanism of action of cucurbitacins in the CTCL cell line, the precise role of JAK2 inhibition remains unknown. Our results, however, hint towards a more prominent role for STAT3 inhibition than JAK2 inhibition. When comparing the effects of the cucurbitacins to those of Stattic and AZD1480, the cucurbitacins appear to have a similar effect to the Stattic. While AZD1480 affected activated STAT5 levels to a similar degree to activated STAT3 levels, Stattic significantly downregulated activated STAT3 levels, but only showed a trend towards a decrease of activated STAT5. This was expected, since Stattic selectively inhibits STAT3 dimerization relative to other STAT family members [61]. The similarity of the effect of the cucurbitacins and Stattic implies a more prominent role for STAT3 inhibition than JAK2 inhibition in the mechanism of action of cucurbitacins in the SeAx cell line.

Since the majority of our experiments were performed in CTCL cell lines, we further confirmed our results by performing a WST-1 assay on primary patient cells. Interestingly, while the concentration of cucurbitacin E and I in this experiment was higher than the IC_50_ values found in the CTCL cell lines, we found a smaller effect on cell viability. This might be because primary patient CD4^+^ cells contain a mixture of healthy and CTCL cells. Moreover, further studies are needed to investigate the extent to which cucurbitacins could be beneficial in the clinic. According to the National Cancer Institute (NCI) guidelines, a IC_50_ value of below 4 μg/ml suggests that a compound might exhibit anti-cancer properties [62]. Our study, however, found values ranging from 6 μg/ml to 12 μg/ml. However, it has been shown that the combination of cucurbitacins with standard anticancer drugs produces synergistic effects. For cucurbitacin E, Sadzuka et al. [63] showed that the combination of cucurbitacin E with doxorubicin resulted in effective cytotoxicity for tumor cells in culture and in vivo. Thus, even though cucurbitacins by themselves might not provide enough cytotoxic potential at maximum safe doses, their combination with other drugs might do. Thus, although more research is needed, cucurbitacins could be a potential therapeutic agent for CTCL.

Our study provides a first impression of the potential of cucurbitacins to treat CTCL. It would be interesting to compare the effect of cucurbitacins on additional cell lines and patient derived cells. Notably, the JAK/STAT pathway has also been implicated in other T-cell lymphomas [27,28]; this would suggest that cucurbitacins could be beneficial in their treatment. Notably, when treating Jurkat cells, a T-cell leukemia cell line, with cucurbitacin E and I, the IC_50_ values were considerably lower than in the CTCL cell lines (see Supplementary Fig. S2). It could be valuable to explore the underlying mechanism for this difference.

While we tested both cucurbitacin E and I, there is a plethora of cucurbitacins with different side chain structures and effects which have not been tested yet in CTCL. Cucurbitacin Q, for example, inhibits the activation of STAT3 but not JAK2 [33] and cucurbitacin An inhibits only JAK2 [33]. These cucurbitacins could provide novel insights into the pathogenesis of CTCL and the mechanism of action of cucurbitacins in these diseases. These natural cucurbitacins, however, have a limited commercial availability. One way this challenge could be overcome is by creating new-to-nature cucurbitacins. This option has become increasingly realistic. Moreover, investigating new-to-nature cucurbitacins could further elucidate the mechanism of action and the effect of different sidechain variations of cucurbitacins in CTCL. This knowledge could aid in creating novel cucurbitacins with higher cytotoxicities, but fewer side effects.

In summary, we show that cucurbitacin E and I induce apoptosis in CTCL cell lines and decrease viability of primary Sz cells. Our results suggest that STAT3 inhibition plays a prominent role in this effect. The precise mechanism of action of cucurbitacins in CTCL, however, remains unclear. A role of STAT5 and JAK2 cannot be excluded and should be explored in future studies. This knowledge could contribute to the development of more effective therapies for CTCL and potentially for other T-cell lymphomas involving dysfunction of the JAK/STAT pathway. To conclude, based on our study, targeting the JAK/STAT pathway through treatment with cucurbitacins is a promising effort which could greatly benefit CTCL patients.

## Declaration of competing interest

The authors state no conflict of interest. This research did not receive any specific grant from funding agencies in the public, commercial, or not-for-profit sectors.
