# Supplementary material for: Cucurbitacin E and I target the JAK/STAT pathway and induce apoptosis in Sézary cells
Source: Biochem Biophys Rep. 2020 Oct 15;24:100832. doi: 10.1016/j.bbrep.2020.100832 (PMC7569298; doi:10.1016/j.bbrep.2020.100832)
Supplement: Multimedia component 1 [file mmc1.docx]

*Supplementary figure 1*


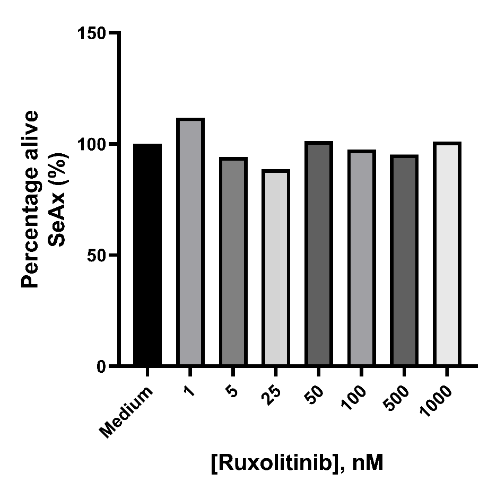

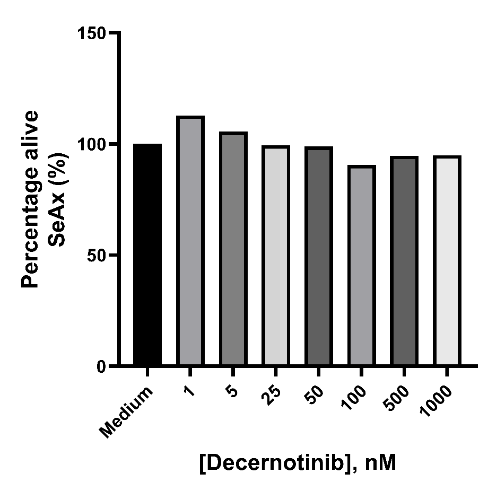

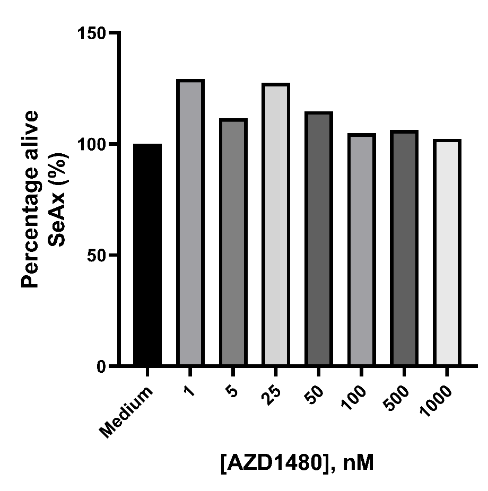


**A**

**B**

**C**

**D**


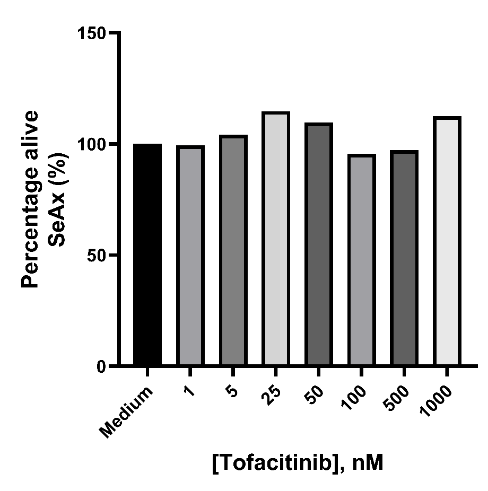


**Supplementary Figure 1 WST-1 cell proliferation assay of AZD1480, decernotinib, ruxolitinib and tofacitinib at low concentrations. A-D:** SeAx cells were seeded at a cell density of 50,000 cells per well in a 96 wells plate. After incubation for 48 hours with a selection of JAKi’s at concentrations ranging from 5 nM to 1 μM, viability was quantitated.

*Supplementary figure 2*


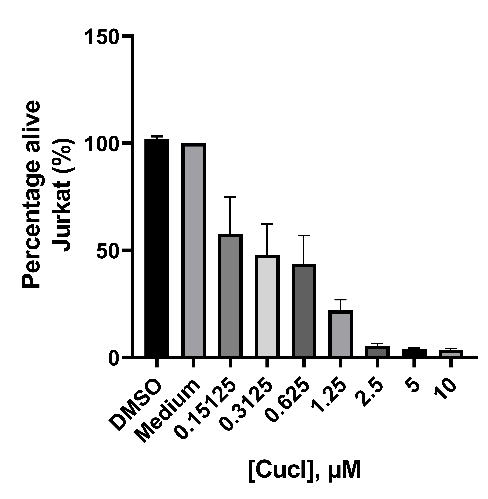

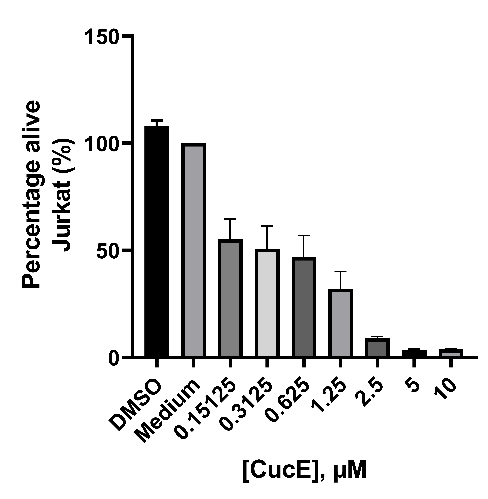


**A**

**B**

**Supplementary Figure 2 WST-1 cell proliferation assay of cucurbitacin E and I in Jurkat cells. A, B:** Jurkat cells were seeded at a cell density of 50,000 cells per well in a 96 wells plate. After incubation for 48 hours with cucurbitacin E and I, viability was quantitated. Cucurbitacin E and I showed IC_50_ values of 0.33 and 0.31 µM respectively. Error bars represent SD of three separate experiments.
